# Effect of Trichomonacide 6-Nitro-1*H*-benzimidazole Derivative Compounds on Expression Level of Metabolic Genes in *Trichomonas vaginalis*

**DOI:** 10.3390/ijms25084568

**Published:** 2024-04-22

**Authors:** Jocelyn Yamin Gutiérrez-Cardona, Ernesto Calderón-Jaimes, Daniel Ortega-Cuellar, Adrián Sánchez-Carrillo, Rosa Angélica Castillo-Rodríguez, Luis Miguel Canseco-Ávila, Luz María Rocha-Ramírez, Víctor Martínez-Rosas, Saúl Gómez-Manzo, Beatriz Hernández-Ochoa

**Affiliations:** 1 Laboratorio de Inmunoquímica, Hospital Infantil de México Federico Gómez, Secretaría de Salud, Mexico City 06720, Mexico; joya3999@gmail.com (J.Y.G.-C.); ecalderj5@yahoo.com.mx (E.C.-J.); ausbir@yahoo.com.mx (A.S.-C.); 2 Laboratorio de Nutrición Experimental, Instituto Nacional de Pediatría, Secretaría de Salud, Mexico City 04530, Mexico; dortegadan@gmail.com; 3 Centro de Investigacion en Ciencia Aplicada y Tecnología Avanzada (CICATA) Unidad Morelos, Instituto Politécnico Nacional, Boulevard de la Tecnología, 1036 Z-1, P 2/2, Atlacholoaya 62790, Mexico; asor108@gmail.com; 4 Facultad de Ciencias Químicas, Campus IV, Universidad Autónoma de Chiapas, Tapachula City 30580, Mexico; cansecoavila@gmail.com; 5 Unidad de Investigación en Enfermedades Infecciosas, Hospital Infantil de México Federico Gómez, Dr. Márquez No. 162, Colonia Doctores, Mexico City 06720, Mexico; luzmrr7@yahoo.com.mx; 6 Laboratorio de Bioquímica Genética, Instituto Nacional de Pediatría, Secretaría de Salud, Mexico City 04530, Mexico; ing_vicmr@hotmail.com

**Keywords:** trichomoniasis, benzimidazole, metabolism, reference gene, trichomonacidal drugs

## Abstract

The parasite *Trichomonas vaginalis* is the etiologic agent of trichomoniasis, the most common non-viral sexually transmitted disease worldwide. This infection often remains asymptomatic and is related to several health complications. The traditional treatment for trichomoniasis is the use of drugs of the 5-nitroimidazole family, such as metronidazole; however, scientific reports indicate an increasing number of drug-resistant strains. Benzimidazole derivatives could offer an alternative in the search for new anti-trichomonas drugs. In this sense, two attractive candidates are the compounds **O_2_N-BZM7** and **O_2_N-BZM9** (1*H*-benzimidazole derivatives), since, through in vitro tests, they have shown a higher trichomonacide activity. In this study, we determined the effect on the expression level of metabolic genes in *T. vaginalis*. The results show that genes involved in redox balance (*NADHOX*, *G6PD::6PGL*) are overexpressed, as well as the gene that participates in the first reaction of glycolysis (*CK*); on the other hand, structural genes such as *ACT* and *TUB* are decreased in expression in trophozoites treated with the compound **O_2_N-BZM9**, which would probably affect its morphology, motility and virulence. These results align with the trichomonacidal activity of the compounds, with benzimidazole **O_2_N-BZM9** being the most potent, with an IC_50_ value of 4.8 μM. These results are promising for potential future therapeutic applications.

## 1. Introduction

*Trichomonas vaginalis* (*T. vaginalis*) is a flagellate parasite that causes trichomoniasis, a sexually transmitted disease (STD) of worldwide importance, reporting 276 million new cases annually. Trichomoniasis is the most common nonviral STD [1], even more common than chlamydia, gonorrhea, and syphilis infections combined [2,3]. *T. vaginalis* is an extracellular parasite and resides in the urogenital tract of both women and men; it can cause vaginitis and urethritis, respectively. However, acute infections are associated with pelvic inflammatory disease, increased risks of human immunodeficiency virus (HIV) infection, and adverse pregnancy outcomes. The first-line treatment against trichomoniasis, in most countries, is based on 5-nitroimidazole drugs such as metronidazole (MTZ) and tinidazole (TNZ) [4,5,6]. This family of drugs is highly effective against most microaerophilic or anaerobic microorganisms. In 1959, MTZ was the first 5-nitroimidazole introduced as a treatment for *T. vaginalis* infection [7]. However, just three years later, the first failure in treatment with this drug was reported [8]. Later, in 2004, TNZ was approved in the USA for the treatment of *T. vaginalis* infection, itself belonging to the 5-nitroimidazole family of drugs [9,10]. 

To date, 5-nitroimidazoles are the only oral medications with demonstrated trichomonacidal activity and that have been approved by the Food and Drug Administration (FDA) [11]. When the standard MTZ treatment fails, there are two alternatives: receiving a higher, often toxic dose of MTZ [12,13], or changing to TNZ, which is not easily accessible for all patients due to availability within countries and costs [14,15]. In addition, allergy to MTZ or TNZ can preclude their use in certain persons with trichomoniasis; allergic reactions such as Stevens–Johnson syndrome or anaphylaxis can occur in response to 5-nitroimidazoles [16,17,18]. The limited catalog of drugs available to treat trichomoniasis infections makes it necessary to search for new compounds with trichomonacidal activity, and better treatment options are urgently needed for patients infected with resistant strains to metronidazole or persons with hypersensitivity to the 5-nitroimidazole drugs.

In this way, the benzimidazole (1*H*-benzimidazole or 1,3-Benzodiazole) scaffold is a privileged structure in new drug design and discovery; it is a nitrogen-containing heterocyclic compound consisting of benzene and imidazole rings, which have gained significance in the field of medicinal chemistry due to their extensive range of pharmacological activities, such as antibacterial, anti-inflammatory, antiviral and antiprotozoal, among others [19]. However, the antiprotozoal properties of this group have not been extensively studied. In this sense, our research group previously reported the synthesis and trichomonacidal activity of two compounds derived from 1*H*-benzimidazole named **O_2_N-BZM7** and **O_2_N-BZM9** [20]. Besides this, it was also discovered that these benzimidazole compounds inhibit the recombinant and bifunctional glucose-6-phosphate dehydrogenase-6-phosphogluconolactonase (G6PD::6PGL) enzyme of *T. vaginalis*. The therapeutic mechanisms of action of the cited compounds are still undefined or only partially known. Therefore, a better understanding of the mechanisms of action of compounds **O_2_N-BZM7** and **O_2_N-BZM9** in *T. vaginalis* is necessary. In this work, we evaluate the effects of compounds on *T. vaginalis* metabolism in culture, as well as the time required to exert its trichomonacidal effect. Moreover, the genetic expression patterns *in T. vaginalis* cultures exposed to compounds **O_2_N-BZM7** and **O_2_N-BZM9** are evaluated against trophozoites exposed to MTZ and trophozoites without exposure to compounds.

## 2. Results and Discussion

### 2.1. Activity Trichomonacidal of Benzimidazole Derivative Compounds

**Figure 1:**
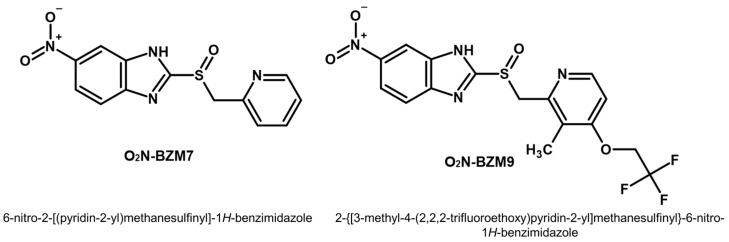
Chemical structure of benzimidazole derivatives (**O_2_N-BZM7** and **O_2_N-BZM9**) evaluated against *T. vaginalis.* The structures were generated in ACD/ChemSketch Freeware 2020.2.0.

Previous work in our research group identified two potent trichomonacide compounds, benzimidazole derivatives 6-nitro-2-[(pyridin-2-yl)methanesulfinyl]-1*H*-benzimidazole and 2-{[3-methyl-4-(2,2,2-trifluoroethoxy)pyridin-2-yl]methanesulfinyl}-6-nitro-1*H*-benzimidazole [20], which are named here **O_2_N-BZM7** and **O_2_N-BZM9**, respectively (Figure 1).

**Figure 2:**
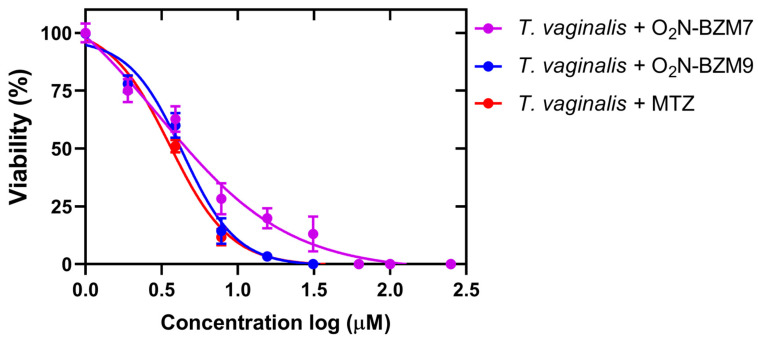
In vitro trichomonacidal activity of benzimidazole-derived compounds. IC_50_ values for antiparasitic activities of **O_2_N-BZM7**, **O_2_N-BZM9,** and MTZ. Values were obtained in three independent assays. Error bars represent S.D. between replicates, and standard errors were lower than 5%.

To evaluate the trichomonacidal effects of benzimidazole derivative compounds **O_2_N-BZM7** and **O_2_N-BZM9**, we assessed the survival of *T. vaginalis* that was cultivated in a medium containing the compounds and **MTZ** (positive control). The results show that both compounds **O_2_N-BZM7** and **O_2_N-BZM9** reduced the trophozoites viability by 100% at 15 μM after 24 h incubation; this same behavior was observed with the drug metronidazole, suggesting that these benzimidazole derivatives could be used as potential antiparasitics. Besides this, it is interesting to mention that the trophozoites treated with 3.9 μM of **O_2_N-BZM7** and **O_2_N-BZM9** decreased *T. vaginalis* viability by 63% and 60%, respectively (Figure 2). Moreover, both compounds reduced the trophozoites viability by >80% at 7.8 μM after 24 h exposure, exhibiting a concentration-dependent inhibition, with an IC_50_ value of 5.8 μM for **O_2_N-BZM7** and 3.8 μM for **O_2_N-BZM9**. As expected, the treatment with a diluent of 0.6% DMSO and negative controls did not induce any reduction in viability; the trophozoites showed negative staining with trypan blue and displayed good morphology and motility. On the other hand, the **MTZ** treatment completely eliminated the viability and exhibited positive trypan blue staining with an IC_50_ value of 3.5 μM.

**O_2_N-BZM9** was 1.6-fold more effective at killing *T. vaginalis* than **O_2_N-BZM7.** This more significant effect could be related to the chemical structures of the compounds; the **O_2_N-BZM9** compound contains a fluorinated functional group binding to pyridine heterocyclic (trifluoroethoxy –OCH_2_CF_3_), which probably improved its trichomonicidal activity. It has been reported that the inclusion of fluorine atoms in pharmaceutical products increases the potency, selectivity, metabolic stability, and pharmacokinetics of the drugs, which are called fluoro-pharmaceutical compounds [21,22]. The successes related to using the fluoro-pharmaceutical compounds have been related to the physicochemical properties of the C–F bond [23], such as high bond strength, polarity, and the minimal steric hindrance of the fluorine atom. In medicinal chemistry, incorporating fluorine into therapeutic drug candidates significantly enhances their biological activities compared to non-fluorinated molecules [24]. For example, in a study by Soria-Arteche et al. [25], a group of benzimidazole-based compounds were synthesized, and it was found that the compound 6-chloro-1-methyl-*N*-(5-nitrothiazol-2-yl)-2-(trifluoromethyl)-1*H*-benzo[d]imidazole-5-carboxamide induced high trichomonicidal activity (IC_50_ = 0.041 μM); besides this, this activity was increased by the addition of a trifluoromethyl group at the 2 position of the benzimidazole ring [25]. In this way, given the IC_50_ results obtained for drug candidates **O_2_N-BZM7** and **O_2_N-BZM9**, it is clear that the activity of fluoro-compound **O_2_N-BZM9** is better than that of **O_2_N-BZM7**.

### 2.2. Kinetic Growth of Trichomonas vaginalis

**Figure 3:**
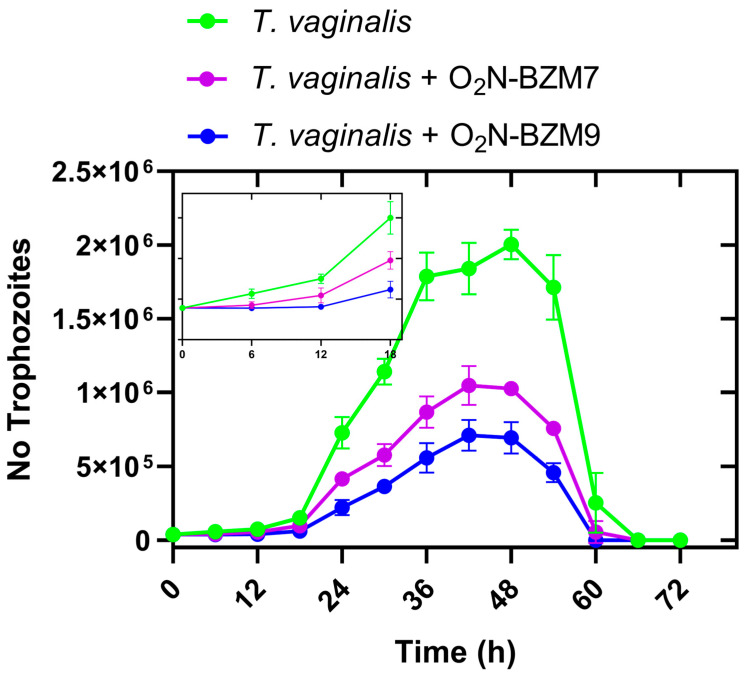
Kinetic growth curve of *Trichomonas vaginalis*. The trophozoites were cultured in the absence (negative control: untreated trophozoites) and presence of each one of the compounds **O_2_N-BZM7** and **O_2_N-BZM9** at their IC_50_ values.

The data obtained from the kinetic growth curve show that both compounds tested reduced trophozoite viability by >50% in the 24 h of incubation, compared with the negative control (Figure 3). When the trophozoites were counted after 6 h of incubation, it was observed that the compounds negatively affected the proliferation of *T. vaginalis* from this first point of evaluation, since the number of trophozoites was lower compared to the control trophozoites without treatment (Inset, Figure 3). It has, thus, been confirmed that the compound **O_2_N-BZM9** inhibits the growth of *T. vaginalis* more drastically than compound **O_2_N-BZM7**. This effect is exacerbated after 12 h of incubation and is maintained until 48 h (Figure 3). After this time, it can no longer be confirmed that the effect of reduced viability is related to the compounds, since in the trophozoite growth curve without treatment, after 48 h, the number of trophozoites decreased drastically. The next objective of this work was to determine the effects of the compounds on the expression levels of metabolic genes in *T. vaginalis*; thus, based on the results of the growth kinetics, we set a 24 h incubation time to carry out the following assays.

### 2.3. Selection of Reference Genes for RT-qPCR Analysis in Trichomonas vaginalis

To accurately detect relative gene expression levels in biological samples via reverse transcription–quantitative polymerase chain reaction (RT-qPCR), it is necessary to use reference genes, which must show expression stability. However, it has been shown that the transcription levels of housekeeping genes like tubulin (*TUB*), actin (*ACT*), glyceraldehyde 3-phosphate dehydrogenase (*GAPDH*), and the 18S rRNA gene [26,27] can change significantly under some specific conditions [26,28,29]. Due to the above, in the present work, we compare the expression stabilities of fifteen candidate reference genes (*CK*, *PFK*, *ALDO*, *TPI*, *GAPDH*, *ENOL*, *PK*, *G6PD*, *6PGDH*, *TKT*, *TALDO*, *PFOR*, *NADHOX*, *ACT*, and *TUB*) (Table 1) to identify the most stable one and propose it as a reference gene to measure expression levels in *T. vaginalis* cultures via RT-qPCR.

### 2.4. Evaluation of the Specificity and Efficiency of the Primer Pairs

**Figure 4:**
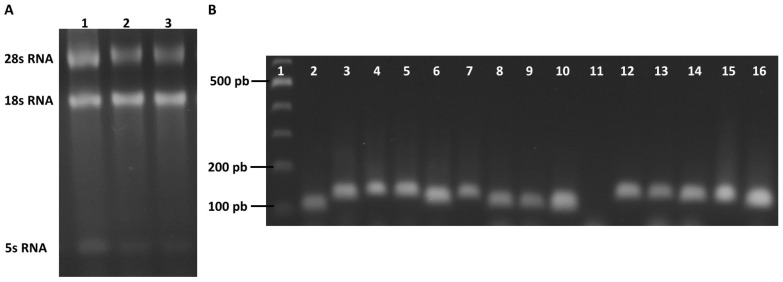
Electrophoresis from total extracted RNA and specificity amplification of the candidate reference genes via PCR endpoint. (**A**) The total extracted RNA was resolved into 5S rRNA, 18S rRNA, and 28S rRNA via electrophoresis. Lane 1: RNA trophozoites without treatment; lane 2: RNA trophozoites with MTZ; and lane 3: RNA trophozoites with **O_2_N-BZM7.** (**B**) Amplification products of the endpoint PCR reaction. Lane 1: molecular weight marker; lane 2: (*CK*) 97 bp; lane 3: (*PFK*) 116 bp; lane 4: (*ALDO*) 125 bp; lane 5: (*TPI*) 122 bp; lane 6: (*GAPDH*) 114 bp; lane 7: (*ENOL*) 124 bp; lane 8: (*PK*) 109 bp; lane 9: (*G6PD*) 109 bp; lane 10: (*6PGDH*) 122 bp; lane 11: (*TKT*) 123 bp; lane 12: (*TALDO*) 123 bp; lane 13: (*PFOR*) 121 bp; lane 14: (*NADHOX*) 118 pb; lane 15: (*ACT*) 123 bp; lane 16 (*TUB*) 115 bp. The gel is representative of three independent experiments.

The quality of the total RNA extracted from the *T. vaginalis* culture was assessed using denaturing electrophoresis. The three bands of 5S rRNA, 18S rRNA, and 28S rRNA were revealed in the gel agarose 2% (Figure 4A). Subsequently, cDNA synthesis was carried out, and the cDNA synthesized from RNA isolated from *T. vaginalis* trophozoites without exposure to compounds was used to validate the efficiency of the primers designed for this study. The endpoint PCR was performed with 100 ng of cDNA; then, the specificity of the primers for candidate genes was evaluated via 2% agarose gel electrophoresis. For genes *CK*, *PFK*, *ALDO*, *TPI*, *GAPDH*, *ENOL*, *PK*, *G6PD*, *6PGDH*, *TALDO*, *PFOR*, *NADHOX*, *ACT*, and *TUB*, a single band of the expected size was obtained; these results indicate that neither primer–dimers nor non-specific amplification products were generated (Figure 4B). For the *TKT* gene, the expected amplification product was not obtained with 100 ng of cDNA (Figure 4B, lane 11). Due to this, the PCR reaction was repeated with a gradient of cDNA concentrations (100–500 ng); however, we were unable to obtain the expected PCR product, so this last gene was eliminated from subsequent studies. 

**Figure 5:**
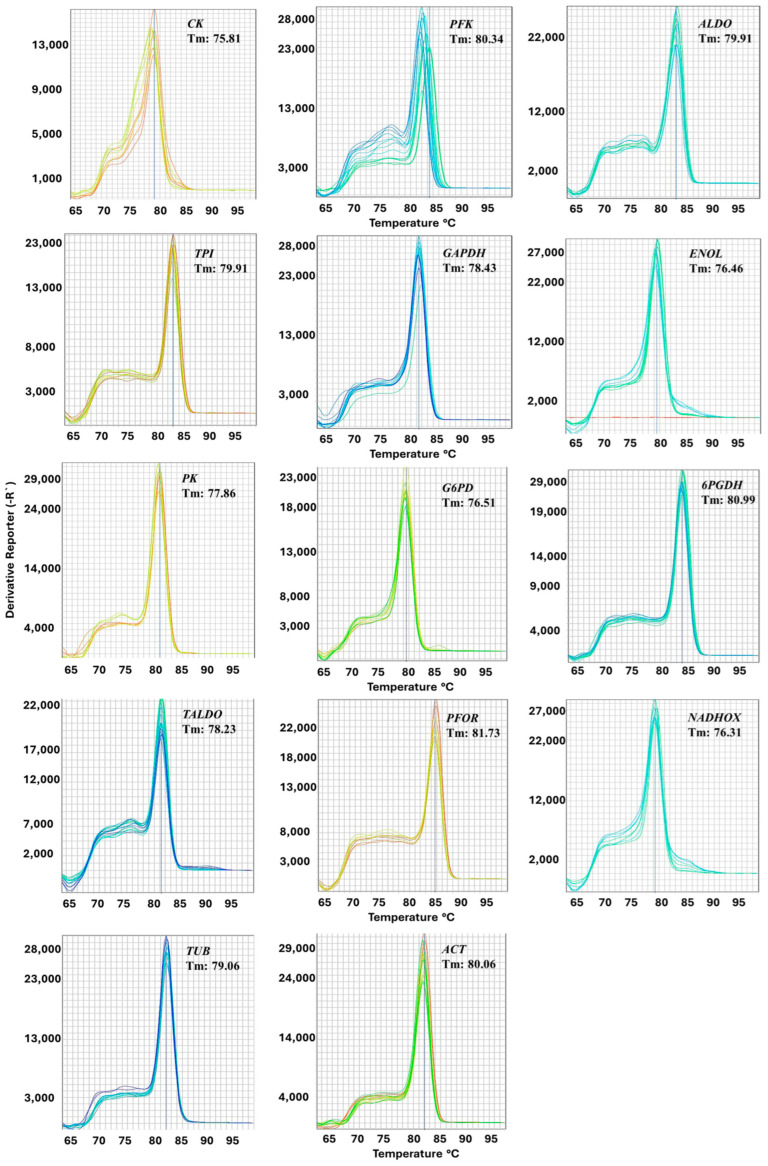
Melting curves of the reference candidate genes. The curves were determined via RT-qPCR; after completing the amplification cycles, the reaction was subjected to a temperature gradient from 60 to 95 °C. Detection was performed with SYBR green, and each of the experiments was performed with 4 independent replicates. The Tm values of the RT-qPCR products of the tested genes are shown in the graphs of the corresponding genes.

Post-amplification melting curve analysis is a way to check real-time PCR reactions for primer–dimer artifacts and to ensure reaction specificity. The melting curve analysis allows us to identify the presence of primer–dimers because they generally exhibit a lower melting temperature than the amplicon, and the primer–dimers reduce PCR efficiency. Therefore, the melting curves were obtained to confirm the specificity of the fourteen primers selected. The results generated for the genes *ALDO*, *TPI*, *GAPDH*, *ENOL*, *PK*, *G6PD*, *6PGDH*, *TALDO*, *PFOR*, *NADHOX*, *ACT*, and *TUB* show a single well-defined peak, indicating that no primer–dimers or unexpected amplicons were observed; however, this was not observed with the *CK* gene, where a secondary peak was observed (Figure 5), so this last gene was eliminated from subsequent studies. The Tm value from the genes ranged from 73.31 °C (*G6PD*) to 81.73 °C (*PFOR*). Subsequently, to determine the correlation coefficient (R^2^), the DNA calibration curves of the five-fold dilution series were used for each candidate reference gene, and the amplification efficiency (E) of each primer pair was evaluated; the parameters obtained by endpoint PCR and RT-qPCR are shown in Table 1. The R^2^ values ranged from 0.92 to 0.998, with all values being greater than 0.998 for *ACT* and *TUB*, and the E values ranged from 90.3% for *GAPDH* to 118% for *NADHOX*. The efficiency values obtained in this study for the primers are within the values established in the literature, indicating that the desirable range for the PCR efficiencies calculated by serial dilution experiments of standard curves should be 90% to 110%, correlating with a slope between −3.1 and −3.58 [30,31,32].

### 2.5. Expression Stabilities of Candidate Reference Genes

**Figure 6:**
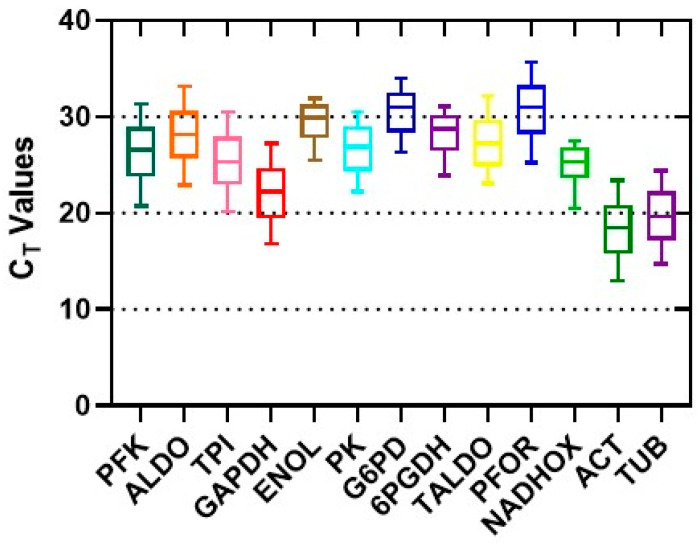
The distribution of Ct values of the 13 candidate reference genes generated via qRT-PCR in *T. vaginalis*. The Ct values for each reference gene were tested in all samples. The boxes indicate the 25th and 75th percentiles, and the lines in the center of the boxes represent the medians. The upper and lower horizontal lines indicate the maximum and minimum values, respectively, and the small squares represent the average values.

To select the ideal reference gene for the normalization of the gene expression assays in *T. vaginalis*, the raw Ct values were used to measure the expression levels of the thirteen candidate genes. The expression levels of candidate reference genes are presented in Figure 6. Diverse levels of mRNA copy number were observed for these genes, with the Ct values ranging from 18 cycles (*ACT*) to 32 cycles (*PFOR*); accordingly, the *ACT* gene presented the highest expression level, whereas the *PFOR* gene had the lowest level. The Ct values were the lowest for *ACT*, *TUB*, *TPI*, and *GAPDH* genes, indicating higher expression levels. By contrast, the *PFOR*, *G6PD*, and *ENOL* genes showed the highest Ct value and, consequently, lower levels of expression.

Subsequently, an evaluation and comparison of the standard deviations of gene expression was carried out using the BestKeeper tool (https://www.ciidirsinaloa.com.mx/RefFinder-master/, accessed on 15 October 2023). The *NADHOX*, *ENOL*, *G6PD*, *6PGDH*, and *PK* genes indicated less variation based on the calculated deviations (lower dispersion Ct), while the *GAPDH*, *ACT*, and *PFK* genes had larger dispersion values (Table 2). Afterward, to evaluate the stability of the expression of the thirteen genes proposed as candidates, we used four statical algorithms—the comparative ΔCt method, NormFinder, geNorm, and RefFinder—to rank the candidate reference genes according to their expression stability [30,31,32].

The geNorm analysis was used to calculate the expression stability value (M) for each candidate reference gene; a low M value indicates more stable gene expression. In the set of 13 candidate genes, all showed an M value less than 1, while *ACT* (0.234), *GAPDH* (0.234), *TUB* (0.289), *TPI* (0.320), *ALDO* (0.337), and *PK* (0.480) had the lowest M values. In contrast, the M value of *NADHOX* was the highest (0.897), suggesting that *ACT*, *GAPDH*, *TUB*, *TPI*, *ALDO*, and *PK* present the most stable expressions. The stabilities of the thirteen candidate reference genes were also calculated with NormFinder tool (https://www.ciidirsinaloa.com.mx/RefFinder-master/, accessed on 15 October 2023), and the evaluated expression stabilities are shown in Table 2. Based on the results, the five most stable reference genes, according to NormFinder, were *PK*, *TALDO*, *TUB*, *ALDO*, and *TPI*, while the least stable genes were *NADHOX*, *ENOL*, and 6PGDH. The results derived with the comparative ΔCt method are similar to those of NormFinder, identifying *PK*, *TUB*, *TALDO*, *ALDO*, and *TPI* as more stable genes and *NADHOX*, *ENOL*, and *6PGDH* as less stable genes.

Among all the genes analyzed, *NADHOX*, *ENOL*, and *6PGDH* were classified as the most unstable references by all programs (Table 2). However, we observed that the stability rankings of the thirteen candidate reference genes we selected, obtained via the comparative ΔCt method and NormFinder, were different from the rankings determined via geNorm and BestKeeper; as such, the results for expression stability obtained with the comparative ΔCt, NormFinder and geNorm algorithms were integrated using the RefFinder tool. The *PK* and *TUB* genes were ranked as the most stable, and the *PFK* and *6PGDH* genes were the least stable.

### 2.6. Validation Candidate Reference Genes

**Figure 7:**
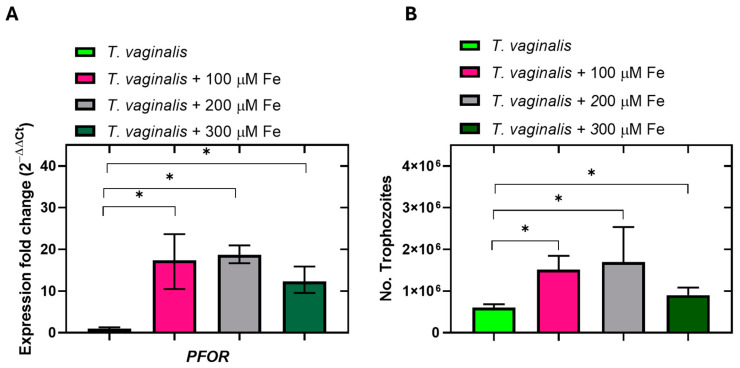
Analysis of *PFOR* gene expression and proliferation of *T. vaginalis* trophozoites treated with ferric ammonium sulfate. (**A**) RT–qPCR for relative quantitation of *PFOR* transcripts under different iron conditions was performed via the 2^−ΔΔCt^ method using 100 ng of template cDNA from parasites grown in media of conventional TYM and three concentrations of NH_4_Fe(SO_4_)_2_, where the *PK* gene was used as a reference. (**B**) Number of trophozoites counted after incubation for 24 h in conventional TYM medium and supplemented with 100, 200, and 300 μM ferric ammonium sulfate. The asterisk indicates a significant difference (*p* < 0.05) in the expression of *PFOR* mRNA under different iron conditions. These experiments were performed in triplicates at least two times with similar results.

Using RefFinder, we were able to select the most stable (*PK*, *TUB*, and *TALDO*) and unstable (*6PGDH*, *ENOL*, and *NADHOX*) candidate internal reference genes in *T. vaginalis*. To verify the feasibility of these internal reference genes, the patterns of expression of the *PFOR* gene in response to exposure to ferric ammonium sulfate were determined, since it has been reported that *PFOR* expression increases with ferric ammonium sulfate treatment [33,34]. The most stable reference gene, *PK*, was selected for the validation assay. With the reference gene *PK*, the overexpression of *PFOR* could be observed when trophozoites were exposed to 100, 200, and 300 μM of NH_4_Fe(SO_4_)_2_, with a significant difference (*p* < 0.05) between the three treatments concerning the trophozoites grown in the conventional medium without ferric ammonium sulfate (Figure 7A). These results correlate with the expression of the *PFOR* protein previously determined by Rivera-Rivas and Rossana Arroyo [34], who determined the expression of the PFOR protein via Western blot, and reported that the anti-TvPFO50r antibody detected a 120 kDa pyruvate–ferredoxin oxidoreductase (PFOR) with greater intensity in the iron-rich condition than in the normal iron and restricted iron conditions [34]. It has been reported that *T. vaginalis* requires high exogenous iron conditions for its survival, metabolism, and proliferation [33,35,36]. We then corroborated the increase in proliferation levels after culturing the trophozoites in increasing concentrations of iron (100, 200 and 300 μM). The results show that with 100 and 200 μM of Fe, the proliferation increased by 2.5- and 2.8-fold in comparison with the conventional TYM medium (Figure 7B). After measuring the expression levels of the *PFOR* gene using *PK* as a reference gene and finding overexpression, we propose *PK* as a new reference gene to measure gene expression levels in *T. vaginalis* cultures.

### 2.7. Level Expression Genes in Trichomonas vaginalis

*T. vaginalis* is a protist that does not contain mitochondria, and in which glycolysis is a dominant metabolic process [37,38], with glucose being the main source of energy. Therefore, some studies on new trichomonacidal drugs have focused on the inhibition of enzymes involved in metabolic pathways that catabolize glucose, for example, glycolysis and the pentose phosphate pathway [39,40,41], since it is hypothesized that disrupting the glucose-catalyzing pathways could result in a reduction in the viability of *T. vaginalis*. Since it was previously reported that compounds **O_2_N-BZM7** and **O_2_N-BZM9** are inhibitors of the recombinant G6PD::6PGL enzyme of *T. vaginalis* [20], it is now of interest to study the effects of the compounds on the expression profiles of the metabolic genes in the parasite.

**Figure 8:**
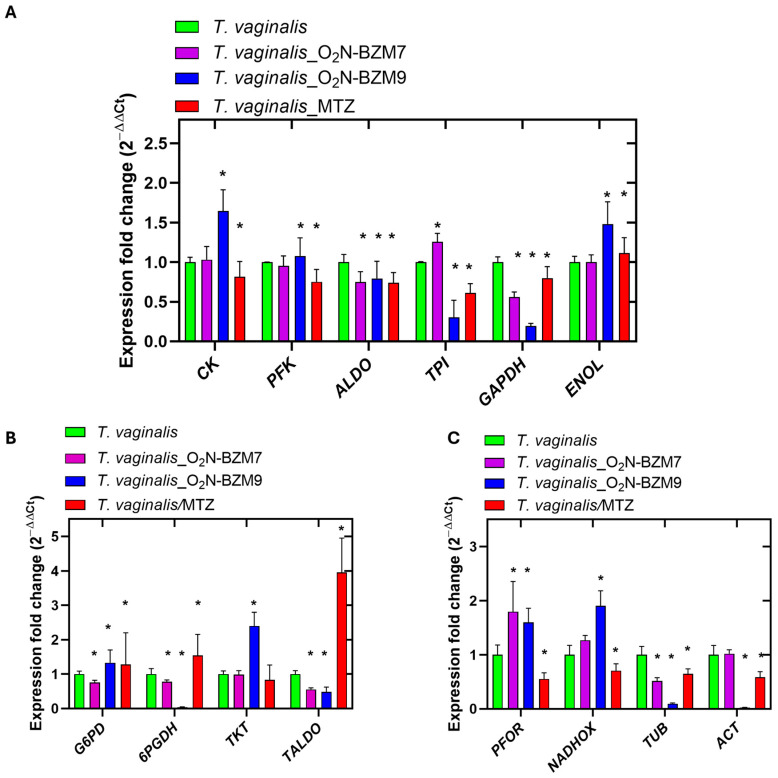
Relative expressions of metabolic and structural genes in *Trichomonas vaginalis* assessed via RT-qPCR. Comparison of gene expression carried out between trophozoites of *T. vaginalis* without treatment used as the negative control, trophozoites of *T. vaginalis* exposed to the compound **O_2_N-BZM7**, and trophozoites of *T. vaginalis* exposed to the compound **O_2_N-BZM9**, using *PK* as a reference gene. (**A**) glycolytic genes. (**B**) Pentose phosphate pathway genes. (**C**) Hydrogenosomal and structural genes. The asterisk indicates a significant difference (*p* < 0.05) in the expression. Error bars indicate ± SD values of three replicates.

A gene expression assay based on RT-qPCR was performed to evaluate the transcription levels of metabolic genes on benzimidazole-derivative-treated *T. vaginalis* trophozoites incubated for 24 h, and trophozoites incubated with 3.5 μM MTZ (IC_50_ value) were used for comparison. The results reveal that among the glycolytic genes that were evaluated, the expression levels of the *ALDO* and *GAPDH* transcripts were significantly reduced after treatments with both **O_2_N-BZM7** and **O_2_N-BZM9** for 24 h (Figure 8A); however, the effect was more substantial with compound **O_2_N-BZM9** than when using *T. vaginalis* without treatment on the expression of *GAPDH*, with a fold change of 0.194 and a 5-fold decrease, respectively. In addition, **O_2_N-BZM9** also exhibited a 3.3-fold reduction in the transcription of the *TPI* gene, reducing the transcript levels to approximately 2-fold lower than that exhibited by MTZ (Figure 8A). On the other hand, **O_2_N-BZM9** significantly increased the *CK* and *ENOL* transcripts in *T. vaginalis*, with fold changes of 1.6 and 1.5, respectively, while after the treatment with **O_2_N-BZM7**, the levels of expression of *CK* and *ENOL* remained the same as in the negative control. Regarding MTZ, it increased the transcription of *CK* 1.2-fold, and *ENOL* showed the same level of expression as *T. vaginalis* without treatment. Finally, we evaluated the expression levels of the *PFK* gene, and no differences were observed between trophozoites exposed to **O_2_N-BZM7** and **O_2_N-BZM9** and those without treatment, while MTZ induced a decrease in the expression of *PFK* (1.3-fold).

In general, MTZ induced a reduction in the expression levels of the glycolytic genes *PFK*, *ALDO*, *TPI*, and *GAPDH* and an increase in the *ENOL* gene, while the *CK* gene was not affected in terms of its expression levels. Between the two benzimidazole compounds, the one that had the greatest effect on expression levels was **O_2_N-BZM9**, which reduced the expressions of *ALDO*, *TPI*, and *GAPDH* and increased *CK* and *ENOL*, while the *PFK* gene remained the same as in the negative control. These results confirm the trichomonacidal activity of the compounds, with benzimidazole **O_2_N-BZM9** being the most potent, with an IC_50_ of 4.8 μM.

**Figure 9:**
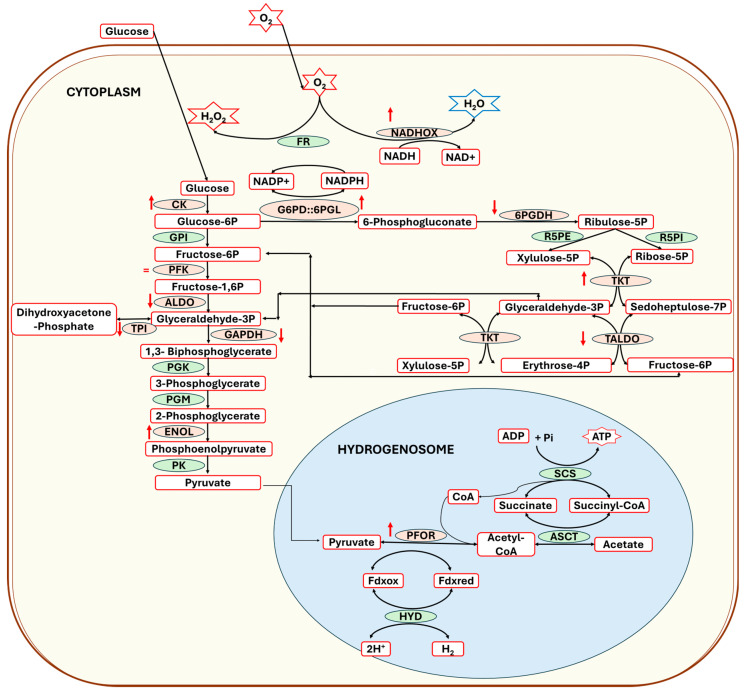
Principal pathways of the energy metabolism, and antioxidant and pentoses phosphate production, in the parasite *Trichomonas vaginalis*. The glucose is catalyzed by glycolysis to form pyruvate, which is passively transported into the hydrogenosome; then, the pyruvate–ferredoxin oxidoreductase (PFOR) enzyme catalyzes the oxidation of pyruvate by iron–sulfur proteins (ferredoxin, Fdx) to produce acetyl-CoA. ATP production follows through a substrate-level phosphorylation catalyzed by the ASCT/SCS cycle, in which two enzymes participate: acetate–succinate CoA-transferase (ASCT) and succinyl-CoA synthetase (SCS). The red arrows ↑ (increased expression) and ↓ (decreased expression).

*T. vaginalis* obtains its energy through the fermentative metabolism of carbohydrates, which involves the oxidation of glucose to produce acetate, adenosine triphosphate (ATP), and molecular hydrogen [42,43,44]. This pathway starts in the cytoplasm, where glucose undergoes glycolysis to form pyruvate, which is passively transported into hydrogenosome (Figure 9), after which the pyruvate–ferredoxin oxidoreductase (PFOR) enzyme catalyzes the oxidation of pyruvate by iron–sulfur proteins (ferredoxin, Fdx) to produce acetyl-CoA. ATP production follows through substrate-level phosphorylation catalyzed by the ASCT/SCS cycle, wherein two enzymes participate: acetate–succinate CoA-transferase (ASCT) and succinyl-CoA synthetase (SCS) (Figure 9). Our results show an overexpression of the gene carbohydrate kinase gene (*CK*)—the first reaction occurring in the preparative phase of glycolysis—in trophozoites exposed to compounds **O_2_N-BZM7** and **O_2_N-BZM9**, while *ALDO*, *TPI*, and *GAPDH* were down-regulated and *PFK* remained unchanged; however, the metabolites (glyceraldehyde-3P, fructose-6P) produced by the enzymes encoded by these genes can be acquired from the pentose phosphate pathway (PPP). The increased expression of *CK*, the enzyme that phosphorylates the glucose to produce glucose-6-phosphate, after **O_2_N-BZM7** and **O_2_N-BZM9** treatment could act like a signal to redirect the metabolic flow towards the PPP, probably due to the parasites requiring an increase in intermediaries to continue proliferation, because it has been reported that glucose metabolism is necessary for the cellular division of *T. vaginalis* [45]. A similar effect was observed for the *ENOL* gene after benzimidazoles were used to treat *T. vaginalis* trophozoites. The ENOL enzyme participates in the penultimate reaction of glycolysis, and its function is the reversible dehydration of 2-phosphoglycerate to phosphoenolpyruvate [46], after which phosphoenolpyruvate is dephosphorylated to produce one molecule of pyruvate and adenosine triphosphate (ATP) via the catalytic reaction of pyruvate kinase (PK); the pyruvate is a necessary component required to continue the production of ATP in hydrogenosome; the overexpression of *ENOL* is probably due to the decrease in ATP, although this must be confirmed experimentally via the measurement of metabolites such as phosphoenolpyruvate, pyruvate, ADP and ATP.

Interestingly, the **O_2_N-BZM9** here manifested a stronger reduction in the transcription of *6PGDH* than in the corresponding trophozoites without treatment (Figure 8B). Notably, the expression of the *6PGDH* gene was almost completely inhibited by **O_2_N-BZM9** at the IC_50_ concentration tested (4.8 μM), while **O_2_N-BZM7** decreased the expression of *6PGDH* 1.3-fold. In contrast, trophozoites treated with **MTZ** exhibited a 1.5-fold increase in gene expression levels of *6PGDH* (Figure 8B). Regarding the levels expression of the enzymes involved in the PPP, we observed a 1.2-fold increase in *G6PD::6PGDL*, which is the gene that codes the first protein of the PPP; this same behavior was observed in trophozoites treated with **O_2_N-BZM9** (1.3-fold increase), while the **O_2_N-BZM7** reduced the expression of *G6PD::6PGDH* 1.3-fold. For trophozoites in the presence of MTZ, a 1.5-fold increase was observed. Regarding the *TKT* and *TALDO* genes, which encode proteins that catalyze reactions in the non-oxidative phase of the PPP, the study revealed that trophozoites exposed to MTZ exhibited a 1.2-fold decrease and a 4-fold increase in *TKT* and *TALDO* transcripts, respectively. At the same time, the compound **O_2_N-BZM9** induces a 2.4-fold increase in *TKT* gene expression, and with the **O_2_N-BZM7** compound, no change in the level of expression was observed. Finally, it is interesting to note that benzimidazole compounds (**O_2_N-BZM7** and **O_2_N-BZM9**) decrease *TALDO* expression by around 1.5-fold. Regarding the levels of expression observed with MTZ, it has been described that MTZ induces oxidative stress in *T. vaginalis*, so the parasites probably increase *G6PD::6PGDH* transcription levels to combat this, since the enzyme G6PD::6PGDH produces a 6-phosphogluconate molecule and NADPH, which serves as a substrate for enzymes that function as O_2_ scavengers, including ferredoxins (FR) (Figure 9). This same behavior was observed in relation to the compound **O_2_N-BZM9**, suggesting it probably also induces oxidative stress in the parasite. With these results, it can be hypothesized that the mechanisms of action of benzimidazole compounds could be different from those of metronidazole.

When trophozoites were exposed to MTZ, they showed 1.8-fold and 1.4-fold reductions in the expression levels of the *PFOR* and *NADHOX* genes, respectively (Figure 8C). These results agree with those previously reported by Leitsch et al. [47], who demonstrated that the activity of NADH oxidase decreases drastically in *T. vaginalis* treated with MTZ [47]. Conversely, the **O_2_N-BZM7** and **O_2_N-BZM9** compounds exhibited an increase in the transcription of *PFOR* and *NADHOX* compared to the corresponding trophozoites without treatment (Figure 8C). *T. vaginalis* contains some enzymes that are key to the elimination of oxygen, which is toxic for this parasite, and thus prevent the deactivation of essential enzymes such as PFOR, as well as hydrogenases such as NADPH oxidase (NADPHOX) and NADHOX [48,49,50], also named flavin reductase enzymes. Since NADHOX has been shown to reduce oxygen to water, its upregulation could contribute significantly to improvements in oxygen scavenging. Apparently, benzimidazole compounds manifest a redox imbalance (increase in reactive oxygen species) in *T. vaginalis*, since the overexpression of the *NADHOX* gene is observed, as well as the *G6PD::6PGL* gene, whose encoded protein participates in the generation of molecules of NADPH, which, through the activity of the enzyme flavin thioredoxin reductase (TrxR) and the nucleotide NADPH, reduce H_2_O_2_ to H_2_O.

Finally, the trophozoites exposed to MTZ showed decreased levels of expression of *ACT* and *TUB* (1.7- and 1.5-fold, respectively); however, once again, the effect of reducing these structural genes was more perceptible in the trophozoites exposed to the compound **O_2_N-BZM9**, which induced 40- and 11-fold reduction in *ACT* and *TUB*, respectively. While compound **O_2_N-BZM7** produced a 2-fold decrease in *TUB* expression, *ACT* expression remained at the same levels as in the negative control. The genes *ACT* and *TUB* encode two structural proteins—actin and tubulin—which have been described to be crucial for morphogenesis, mitosis, and virulence in *T. vaginalis* [51,52,53,54]. Tubulin has been detected in the axostyle, flagella, costa, pelta, and basal body structures that contribute to the parasite’s motility [55]. Actin is essential to *T. vaginalis*’ morphological transformation through the formation of filopodia and pseudopodia, and the attachment to host cells to establish infection (cytoadherence) [51,56]. Therefore, **O_2_N-BZM9** compounds probably principally induce alterations in their motility and morphology, and consequently cause death. Besides this, the parasite’s virulence is also negatively affected by reductions in its cytoadherence.

## 3. Materials and Methods

### 3.1. Parasite and Culture Conditions

The *T. vaginalis* trophozoites (ATCC30001) were cultivated in Diamond’s Trypticase–Yeast–Maltose (TYM) medium, pH 6.0, supplemented with 10% sterile heat-inactivated horse serum and incubated under microaerophilic conditions in screw-capped glass tubes at 37 °C with 5% CO_2_ for 24 h. When the culture reached 90% confluency, the trophozoites were incubated at 4 °C for 15 min, collected by centrifugation at 375× *g* for 5 min and washed with sterile PBS (15 mM phosphate buffer and 154 mM NaCl, pH 7.0); then, the viability, motility and morphology of the parasites were evaluated under light microscopy and by the trypan blue (0.4%) exclusion assay to ensure that minimum viability of 95% had been reached before proceeding to trichomonacidal assays.

### 3.2. Trichomonacidal Activity of Compounds

Drug susceptibility assays were performed to evaluate the trichomonacidal potential of compounds derived from 1*H*-benzimidazole (**O_2_N-BZM7** and **O_2_N-BZM9**) as previously described [20]. For the assay, tubes of 1.5 mL were seeded with an initial density of 2.6 × 10^4^ trophozoites/mL on TYM. Then, **O_2_N-BZM7** and **O_2_N-BZM9** that had been previously diluted in dimethylsulfoxide (DMSO) were added into the tubes at different concentrations (0, 2, 5, 10, and 50 μM), and the tubes were incubated at 37 °C with 5% CO_2_. After 24 h of culture, the tubes were incubated for 10 min on ice to detach the trophozoites, and using an aliquot (1:1, *v*/*v*) and trypan blue (0.4%), the trophozoites were counted in a Neubauer chamber and were evaluated for motility, morphology, and viability. Trophozoites cultured without compounds, with 0.6% DMSO (diluent) or metronidazole (3.5 μM), were the negative and positive controls, respectively, for the growth of parasites. The concentrations of the compounds were plotted against the corresponding percentages of inhibition, and the inhibitory concentration 50 (IC_50_) was calculated using GraphPad Prism 8.0.1 software. 

### 3.3. Effects of Compounds on Trichomonas vaginalis Growth Kinetic

To ascertain the time required for the compounds to exert antiparasitic activity against *T. vaginalis*, kinetic growth curves of the trophozoites were developed in the absence and presence of the compounds. Once more, the 1.5 mL tubes were prepared according to the methodology above. The IC_50_ of each compound was added to each tube and incubated at 37 °C in 5% CO_2_ for 96 h. The viability and number of trophozoites were monitored under an optical microscope at 6, 12, 18, 24, 48, 72, and 96 h using the trypan blue dye exclusion test (0.4%). Trophozoite viability for cultures of trophozoites without the compound was 100%. The assays were carried out independently in triplicate.

### 3.4. Validation and Selection of Reference Genes for RT-qPCR Analysis in Trichomonas vaginalis

#### 3.4.1. Primer Design

Fifteen candidate genes were evaluated for their applicability as a reference in RT-qPCR assays to determine gene expression profiles in *T. vaginalis* cultures (Table 3). The sequences for primer design were obtained from GenBank and then compared to the sequences reported in the Trichomonas genome database (https://trichdb.org/trichdb/app//, accessed on 15 January 2023). The primer pairs were designed based on mRNA sequences of different genes using the program Primer3 (http://primer3.ut.ee, accessed on 15 January 2023) with the following parameters: length 18–22 bp, GC content 45 to 55%, Tm 60 ± 2 °C, and product size 60 to 120 bp. The primer pairs were also evaluated for primer–dimer formation using the OligoEvaluator™ tool (http://www.oligoevaluator.com/AddUser.jsp, accessed on 15 January 2023).

#### 3.4.2. PCR Efficiency and Specificity

The specificities of the primer pairs were verified by endpoint PCR, using cDNA from *T. vaginalis* trophozoites as the template and the enzyme Q5™High-Fidelity DNA polymerase (New England, BioLabsinc, Beverly, MA, USA), with the following amplification conditions: 30 s at 95 °C; 30 cycles of 10 s at 95 °C, 30 s at 61 °C, and 30 s at 72 °C; finally, 10 min at 72 °C. The amplified fragments were separated by electrophoresis in a 2% (*w*/*v*) agarose gel, dyed with GelRed (Nucleic Acid Gel, Biotium, Fremont, CA, USA), and visualized on a MultiDoc-It (UVP). To confirm the specificity of the primers and determine the efficiency of the PCR reaction, RT-qPCR analysis was carried out on a StepOne™ Real-Time PCR System and a Fast SYBR^®^ Green Master Mix Kit (Applied Biosystems, Foster City, CA, USA), applying a 5-fold serial dilution consisting of five concentrations of cDNA, beginning at 100 ng; we then constructed standard curves to determine amplification efficiencies (E) for each candidate reference gene. Once the reaction cycles were completed, the melting curves for each gene were obtained, and the reactions were heated in a temperature range of 60 °C to 95 °C. 

#### 3.4.3. Validating Candidate Reference Genes

The objective of this assay was to identify a gene that presents constant and invariant expression in all samples, such that its expression cannot be modified by the study conditions or by experimental treatments. For this analysis, the Ct values obtained for each of the candidate reference genes were used to monitor the stability of the genes via four statistical methods: Normfinder [57], Genorm [58], and the comparative delta–Ct method [59]. In this way, the best genes were identified and referenced for data normalization in RT-qPCR analysis. It is essential to mention that the rankings of reference genes tested using different algorithms may change. To resolve this issue, we used RefFinder [60], an analysis program that employs algorithms to comprehensively evaluate and classify reference genes based on experimental data.

#### 3.4.4. RNA Extraction, cDNA Synthesis, and Quantitative PCR (qPCR) 

Total RNA was extracted from *T. vaginalis* parasites treated with compounds **O_2_N-BZM7** and **O_2_N-BZM9** (5.8 μM and 3.8 μM, respectively) for 24 h, respectively, using the Trizol reactive agent (Thermo Fisher Scientific, Waltham, MA, USA). RNA contamination and degradation were monitored on 2% agarose gels, and RNA purity and concentration were measured with a NanoPhotometer^®^ spectrophotometer (IMPLEN, Westlake Village, CA, USA). Reverse transcription (RT) was carried out using Oligo dT_18_ for first-strand synthesis (Thermo Fisher Scientific, Waltham, MA, USA) and reverse transcriptase (Thermo Fisher Scientific, Waltham, MA, USA). RT-qPCR using the SYBR Green qPCR Master Mix (Bio-Rad, Hercules, CA, USA) was performed on a One-Step Real-Time PCR system (Applied Biosystems). The *PK* gene was used as an internal control for the normalization of gene expression in all experimental groups. 

### 3.5. Probit Analyses

Probit analyses were performed using the data on *T. vaginalis* growth with different concentrations of the compounds and metronidazole. This permitted the calculation of IC_50_ values. We used the GraphPad Prism 4 package to perform the one-way analysis of variance for data. 

## 4. Conclusions

The compounds **O_2_N-BZM7** and **O_2_N-BZM9** showed good trichomonicidal activity on trophozoites, with **O_2_N-BZM9** being the most potent, showing a low IC_50_ value similar to those of the reference MTZ drugs (3.8 and 3.5 µM, respectively), the compound could be effective at low concentrations and therefore show lower systemic toxicity when administered to the patient. The compound **O_2_N-BZM9** has fluorine atoms in its structure, which likely improves its antiparasitic activity. Regarding the expression profiles of metabolic genes, genes involved in redox balance (*NADHOX*, *G6PD::6PGL*) were overexpressed, as well as the gene that participates in the first reaction of glycolysis (*CK*). On the other hand, structural genes such as *ACT* and *TUB* showed decreased expression in trophozoites treated with the compound **O_2_N-BZM9**, which could affect their morphology, motility, and virulence. These results are promising for potential future therapeutic applications of compounds **O_2_N-BZM7** and **O_2_N-BZM9,** such as trichomonacidal drugs.

## Figures and Tables

**Table 1:** Primers and their parameters obtained via endpoint PCR and RT-qPCR.

Gen Symbol	5′-3′Sequence	Amplicon Size	Tm	RT-qPCR E (%)	Slope	R^2^
*CK*	FW: ACAACAGGAGCCGGAGATG RV: AGCAGCACAACCTCTCTTTG	97	59.18 58.40	ND	ND	ND
*PFK*	FW: TGCAGTTCTCTCTAGTGGCC RV: CACGGAAGCCACCAGTAATG	116	59.10 58.91	101	−3.296	0.964
*ALDO*	FW: AAGTCACTCGGTCTCTGCAA RV: TTGACGGAGGCTGTGATGAT	125	58.96 59.1	98.5	−3.356	0.991
*TPI*	FW: GGCAAGTGGGACGATGTTG RV: TTAGCAGCAAGGATGTCACG	122	59.12 58.27	94.4	−3.464	0.995
*GAPDH*	FW: CCAAGTTGTCGCTATCCACG RV: TGCTTAGCCTCATCGACTGT	114	59 58.81	90.3	−3.577	0.996
*ENOL*	FW: ACAGGTGTTGGTGAAGCTCT RV: AGCACATTCCCTTGAGAGCT	124	59.16 59	110	−3.055	0.975
*PK*	FW: CCACAAGCAAACACTCGACA RV: CTCCAACTTGCCAACACGAA	109	59 59	114.8	−3.042	0.989
*G6PD*	FW: ATTCTCACGTCTCCACCAGG RV: GTCATCGTAGCCACCAGAGA	109	59.1 58.9	117	−3.068	0.959
*6PGDH*	FW: CGATGGTGGCAACTCTCACT RV: CTCTTCACCGCCGGAGATAC	122	60 60.1	104	−3.226	0.991
*TALDO*	FW: TCCTCAAGATTGTCCCAGGC RV: TCTTGATTCCGGCTTCGTGA	123	59.3 59.3	111	−3.07	0.994
*ACT*	FW: GTCAAGCTTCTCACAGAGCG RV: GGCCTTCTCCATTTCAGCAT	123	58.9 58.2	90.4	−3.574	0.998
*TUB*	FW: CTTCCGTGGCCGTATGTCAT RV: GCAGATAGCGGACTTGACGT	115	60 60	97.1	−3.392	0.998
*PFOR*	FW: CCAGATCACACCACTCGACT RV: TTCCCAGTTCTTGCCCTCTT	121	59.1 58.9	105	−3.188	0.942
*NADHOX*	FW: ATTGGCTTGGCGTCCTTGAT RV: TCGACGAGAACTGCACCTTC	118	60.3 60.0	118	−3.045	0.92

ND: Not determined.

**Table 2:** Ranking of candidate reference genes in order of expression stability.

GeNorm	M	Comparative ΔCt Method		NormFinder		Ranking	
*ACT*	0.234	*PK*	0.73	*PK*	0.195	*PK*	2.43
*GAPDH*	0.234	*TUB*	0.75	*TALDO*	0.387	*TUB*	3.57
*TUB*	0.289	*TALDO*	0.75	*TUB*	0.490	*TALDO*	3.83
*TPI*	0.320	*ALDO*	0.76	*ALDO*	0.495	*GAPDH*	4.56
*ALDO*	0.337	*TPI*	0.79	*TPI*	0.578	*ACT*	4.98
*TALDO*	0.400	*GAPDH*	0.82	*GAPDH*	0.649	*ALDO*	5.03
*PK*	0.480	*ACT*	0.85	*ACT*	0.690	*TPI*	5.62
*PFK*	0.540	*PFK*	0.93	*PFK*	0.693	*NADHOX*	6.45
*PFOR*	0.616	*PFOR*	0.95	*PFOR*	0.721	*G6PD*	6.84
*G6PD*	0.726	*G6PD*	1.02	*G6PD*	0.828	*ENOL*	7.18
*6PGDH*	0.807	*6PGDH*	1.06	*6PGDH*	0.893	*PFOR*	7.48
*ENOL*	0.861	*ENOL*	1.07	*ENOL*	0.928	*6PGDH*	7.95
*NADHOX*	0.897	*NADHOX*	1.08	*NADHOX*	0.944	*PFK*	8.66

**Table 3:** Genes analyzed in this study.

GenBank Accession Number	Gene Annotation	Gen Symbol	Function
XM_001579622.1	Carbohydrate kinase	*CK*	Kinase in glycolysis
XM_001581728.2	Phosphofructokinase	*PFK*	Kinase in glycolysis
XM_001315350.2	Aldolase IIB	*ALDO*	Oxidoreductase in glycolysis
XM_001320301.2	Triosephosphate isomerase	*TPI*	Isomerase in glycolysis
XM_001581066.2	Glyceraldehyde-3-phosphate dehydrogenase	*GAPDH*	Oxidoreductase in glycolysis
XM_001325471.2	Enolase	*ENOL*	Lyase in glycolysis
XM_001329865.2	Pyruvate kinase	*PK*	Oxidoreductase in glycolysis
XM_001321943.2	Glucose-6-phosphate 1-dehydrogenase	*G6PD*	Oxidoreductase in pentose phosphate
XM_001323727.2	Phosphogluconate dehydrogenase	*6PGDH*	Oxidoreductase in pentose phosphate
XM_001326902.1	Transketolase	*TKT*	Transferase in glycolysis
XM_001330311.2	Transaldolase	*TALDO*	Transferase in glycolysis
XM_001321286.2	Pyruvate–ferredoxin oxidoreductase proprotein	*PFOR*	Oxidoreductase in hydrogenosome
XM_001315387.2	Diflavin flavoprotein A (NADH oxidase)	*NADHOX*	O2-Detoxifying enzyme
XM_001321203.2	Tubulin beta 4 chain	*TUB*	Cytoskeletal structural protein (Flagella, median body, ventral disc)
XM_001301716.2	Actin	*ACT*	Cytoskeletal structural protein (all cell)

## Data Availability

Data are contained within the article.
